# Exosomal miR-21 promotes proliferation, invasion and therapy resistance of colon adenocarcinoma cells through its target PDCD4

**DOI:** 10.1038/s41598-020-65207-6

**Published:** 2020-05-19

**Authors:** Li-Hua Sun, Dan Tian, Ze-Cheng Yang, Jin-Long Li

**Affiliations:** 1 Department of Anesthesiology, The Second Hospital of Jilin University, Changchun, China; 2 Department of Gastrointestinal Surgery, The Second Hospital of Jilin University, Changchun, China

**Keywords:** Cancer microenvironment, Colon cancer

## Abstract

Exosomes contain cell-specific collections of bioactive materials including proteins, lipids, and RNAs that are transported to recipient cells to exert their impacts. MicroRNAs (miRNAs) can function as tumor suppressor or oncogenic genes and miR-21 is one of the most frequently up-regulated miRNAs in solid tumors including colon cancer. The aim of this study was to investigate the role of miR-21, secreted from exosomes, in proliferation and invasion of colon cancer, along with the mechanistic details. We used a variety of biochemical techniques including ultracentrifugation-based exosome purification, electron transmission microscopy, western blot and RT-qPCR to detect the expression levels of miR-21 in exosomes purified from culture media of human colonic adenocarcinoma cell lines. We then performed functional and mechanistic studies using three colon cancer cell lines HT29, T84 and LS174 as well as the normal colon epithelial cells CRL1831. miR-21 target PDCD4 was investigated for its role in mediating miR-21 effects. Expression of miR-21 was significantly up-regulated in exosomes of colon cancer cells, compared to the normal human colon epithelial cells. Treatment of colon cancer cells with isolated exosomes or miR-21 led to an increased expression of genes involved in cell proliferation, invasion and extracellular matrix formation. miR-21 targets PDCD4, TPM1 and PTEN were down-regulated by exosomes and silencing of PDCD4 mimicked miR-21 functional effects, even the induced resistance against 5-FU. Our study suggests that targeted inhibition of exosomes, particularly those carrying miR-21, may represent a novel approach for treatment of colorectal cancer.

## Introduction

Colorectal cancer (CRC) is a frequently diagnosed cancer that is responsible for a large number of deaths, particularly in developed countries^1–3^. Furthermore, the overall five-year survival of CRC patients varies: it is around 8% for stage IV patients and about 93% for early I patients. Presently, surgical resection is the primary course of treatment and chemotherapy is associated with modest survival benefit as well^4^. Despite numerous efforts, the progress towards treatment of CRC has been dismal. Therefore, there is urgent need to validate novel biomarkers for effective management of CRC patients in clinics.

MicroRNAs (miRNAs) are small non-coding RNAs that influence a variety of biological processes^5,6^. Dysregulation of miRNAs has been reported in many cancers. It has been estimated that miRNAs regulate around 30% of all human genes^7^. Further, differential miRNA expressions have been proposed to help classify different human cancers with a role to play in cancer invasion, progression as well as response to therapies^8^. Also, the pro- and anti-cancer activity of miRNAs, both *in vitro* and *in vivo*, has been detailed in many published studies^9,10^. For example, the regulation of miR-21 and its role in carcinogenesis have received an extensive investigation^11–15^.

Cell-to-cell communication is critically important for all living organisms. Cells are known to mutually communicate through different mechanisms, such as, via soluble mediators like hormones, cytokines, and chemokines. The first report on production of exosome-like MVs (membrane-bound microvesicles) by ovarian cancer and neoplasic cell lines appeared almost 40 years ago^16^. Exosomes are small membrane vesicles, approximately 30–100 nm that carry proteins, lipids, mRNAs, and miRNAs^17^. In the last decade, there has been a major interest in understanding MV function and the resulting biological implications^18^. miRNAs are now understood to secreted by many cells types, including cancer cells, into body fluids such as blood, urine, breast milk, and saliva, via exosomes^19,20^.

A previous study has shown that the serum exosomal levels of multiple miRNAs including miR-21 were significantly elevated in primary CRC patients^3^. Combined with a recent study showing that miR-21 overexpression results in changes in the expression of genes involved in cell proliferation and extracellular matrix formation in fibroid cells^14^, in this study, we tested the hypothesis that miR-21 promotes cell proliferation of colon cancer by regulating expression of genes that are involved in ECM formation. We show that miR-21 was significantly upregulated in exosomes purified from colonic cancer cell lines. Furthermore, treatment of these cell liens with isolated exosomes or miR-21 led to an increased expression of these genes, suggesting targeting of exosome secretion for CRC treatment.

## Materialas and Methods

### Cell culture

Human colon carcinoma cell lines HT29, T84, and LS174 were maintained in RPMI-1640 medium supplemented with or without 10% fetal bovine serum. The cells were grown in a humidified incubator at 37 °C and 5% CO_2_. siRNA for silencing of PDCD4 was from Santa Cruz Biotechnology (Shanghai, China).

### Isolation of exosomes from culture media

Exosome fractions were prepared by a step-wise centrifugation-ultracentrifugation method, as described previously with minor modifications^3^. Briefly, culture medium from colon cancer cell lines was centrifuged at 13,000 rpm for 60 min to remove the cell debris. The cleared supernatant was passed through a 0.20 μm filter and then ultracentrifuged at 200,000 g for 60 min using a TLA-110 rotor (Beckmann). The pellets were washed with phosphate-buffered saline (PBS) and then resuspended in PBS as exosome-enriched fractions.

### Preparation of exosome RNAs

The exosomes were mixed with 500 μL of Trizol-LS reagent (Invitrogen) and the aqueous phase was collected after adding chloroform. After the addition of ethanol to the aqueous phase, total RNAs were further purified using RNeasy Mini Spin Columns (Qiagen). The RNA sample was dried and then dissolved in 10 μL of nuclease-free water. The concentration of RNA was determined using a NanoDrop spectrophotometer (Thermo Scientific).

### Quantitative RT-PCR

Total RNAs from cell lines were extracted by Trizol method (Invitrogen, CA, USA). Dried RNA pellets were re-suspended in appropriate volumes of DEPC H_2_O. Total cDNA synthesis was prepared by the miR-21, U6 snRNA-specific or oligo-dT primers method using the Superscript II Reverse Transcription kit (Invitrogen). Quantitative PCR was performed using an ABI7300 machine (Applied Biosystems). miRNA/ mRNA levels were determined with relative to U6 or GAPDH expression using the SYBR Green PCR kit (Qiagen), respectively. Fold change was determined by the method of cycle threshold (CT) in the formula of 2^−ΔΔCT^. U6 snRNA forward, 5′-TGCGGGTGCTCGCTTCGGCAGC-3′. U6 snRNA reverse, 5′-GGGTCCGAGGTGCACTGGATACGACAAAATATGG-3′; GAPDH forward, 5′-CTCCCGCTTCGCTCTCTG-3′, GAPDH reverse, 5′- CTGGCGACGCAAAAGAAG -3′; MMP-2 forward, 5′-TGCTGGAGACAAATTCTGGA-3′, MMP-2 reverse, 5′-TTGGTTCTCCAGCTTCAGGT-3′; MMP-9 forward, 5′-TGGGAAGTACTGGCGATTCT-3′, MMP-9 reverse, 5′-CCTGTGTACACCCACACCTG-3′; MMP-11 forward, 5′-CTTCCGAGGCAGGGACTACT-3′, MMP-11 reverse, 5′-AGAAGTCAGGACCCACGAGA-3′; TIMP-2 forward, 5′-GATGCACATCACCCTCTGTG-3′, TIMP-2 reverse, 5′-GTGCCCGTTGATGTTCTTCT-3′.

As recently described^21^, We used the miScript PCR System (Qiagen) for quantification of mature miRNAs from exosomes with a universal primer and miRNA-specific primer included in the kit according to the manufacture’s protocol. All expression levels were normalized to total RNAs extracted from the purified exosomes.

### Western blot

The exosomes were mixed with 1× SDS loading buffer, boiled at 95 °C for 5 min, and centrifuged. Supernatants were separated on SDS–10% PAGE and transferred onto PVDF membranes (Bio-Rad). Membranes were blocked in 5% nonfat milk in PBST (10 mM phosphate buffer, pH 7.2; 150 mM NaCl; and 0.1% Tween-20) for 60 min, washed three times with PBST, and incubated with CD9 or CD63 antibody (Santa Cruz Biotech, USA, 1:500) at 4 °C overnight. Membranes were incubated with horseradish peroxidase-conjugated secondary antibody at 1:5,000, and developed by Immun-Star HRP Substrate (Bio-Rad, CA, USA).

### Cell proliferation assay

MTT [3-(4, 5-dimethylthiazol-2-yl)-2, 5-diphenyltetrazoliumbr-omide] - based assay was performed to estimate the change in proliferation under differential experimental conditions, as previously described^22,23^. Cells were seeded into 96-well plates (5,000 cells/well in 200 µL medium) and incubated for indicated time periods at 37 °C, 5% CO_2_. Cells were transfected with miR-21 mimics, or anatgomirs using Lipofectamine 2000 Reagent (Life Technologies) as indicated. Cells cultured with complete medium were used as blank control. At the end of incubation, 20 µL of 5 mg/ml MTT (Sigma, USA) solution was added per well, and the cells were incubated for another 4 hr at 37 °C. Supernatants were removed and formazan crystals were dissolved in 150 µL of DMSO (Sigma-Aldrich). Finally, OD was determined at 490 nm using multi-microplate test system (Victor3, MA, USA). In each assay, five parallel wells were made, and the results were collected as the mean of minimum three independent experiments.

### Cell Invasion assay

Cell invasion assay was performed by using the Cultrex^R^ cell migration assay kit purchased from R&D Systems (Shanghai, China), as per the company’s instructions. The assay employs a simplified Boyden chamber-like design that consists of two chambers separated by a filter coated with basement membrane extract (BME). Cells were placed in the top chamber and those cells that invaded through the BME were detected using Calcein AM. Cell dissociation/Calcein AM solution was poured in to the bottom chamber in order to dissociate the invading cells from the filter and for detection using fluorescence as the cells internalized Calcein AM.

### Statistical analysis

Data were expressed as the mean ± standard deviation (SD). We used a Student’s *t*-test to compare two groups. p < 0.05 was considered significantly different.

## Results

### miR-21 is up-regulated in exosomes purified from colon cancer cells

**Figure 1:**
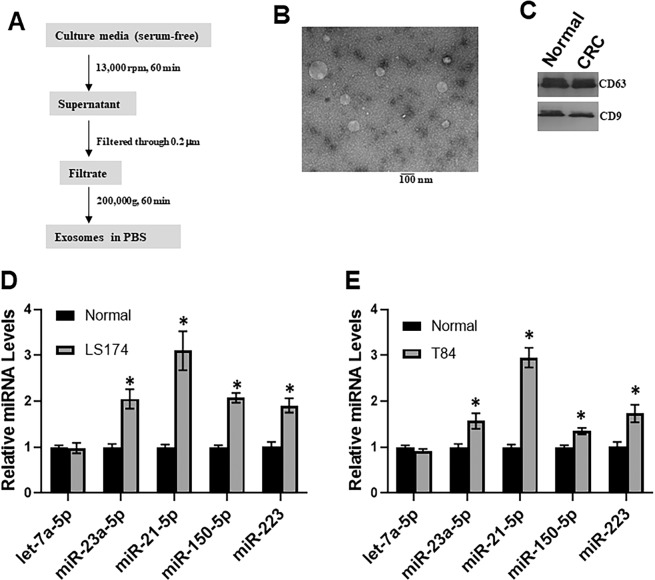
Analysis of exosomes isolated from serum-free culture media of normal and Colon cancer cell lines. (**A**) Isolation procedure of exosomes. (**B**) Imaging of exosomes isolated from the culture media by transmission electron microscope. Bar length = 100 nm. (**C**) Western blot analysis of isolated exosomes. Human exosomal markers CD9 or CD63 were evaluated. (**D**) Quantitation of exosome miRNAs as indicated isolated from ‘normal’ human colon epithelial cell line CRL1831 or CRC cell line LS174 by RT-qPCR (n = 3 individual experiments). (**E**) Quantitation of exosome miRNAs as indicated isolated from ‘normal’ human colon epithelial cell line CRL1831 or CRC cell line T84 by RT-qPCR (n = 3 individual experiments). *p < 0.01.

As previously described^3^, the serum exosomal levels of seven miRNAs including let-7a, miR-1229, miR-1246, miR-150, miR-21, miR-223, and miR-23a, were significantly enriched in primary CRC patients, even those with early stage disease, than in healthy controls. First of all, we used step-wise centrifugation-ultracentrifugation method to purify exosomes from serum-free culture media from colon cancer cell line LS174. We cultured cell with serum-free to prevent any contamination from fetal bovine serum that could contain remarkable amount of exosomes (Fig. 1A). Exosomes are heterogeneous in morphology and size, and typically range between 30 and 100 nm in diameter. The purity of exosomes was further visualized by transmission electron microscopy (Fig. 1B) and Western blot analysis with use of antibodies against well-characterized exosomal markers CD9 and CD63^24^. Thus, we ensured the quality of exosomes through multiple methods.

We used RT-qPCR to further determine levels of miRNAs including let-7a, miR-150, miR-21, miR-223, and miR-23a and found that miR-150, miR-21, miR-223, and miR-23a were significantly (p < 0.01) enriched in exosomes purified from colon cancer cell line LS174, as compared to normal human colon epithelial cell line CRL1831 (Fig. 1D). In particular, the expression of miR-21 was the most increased in cancer cells, compared to the normal cells. In a further validation, using another colon cancer cells, T84 cells (Fig. 1E), we again found a similar trend. A number of miRNAs were found to be significantly (p < 0.01) enriched in exosomes from cancer cells, compared to normal cells, and miR-21 stood out as the most over-expressed miRNA. Taken together, these results support that exosomal miRNA signatures appear to reflect pathological changes of CRC patients, with miR-21 emerging as one of the primary miRNAs.

### Cancer exosomes stimulate expression of ECM genes in colon adenocarcinoma

**Figure 2:**
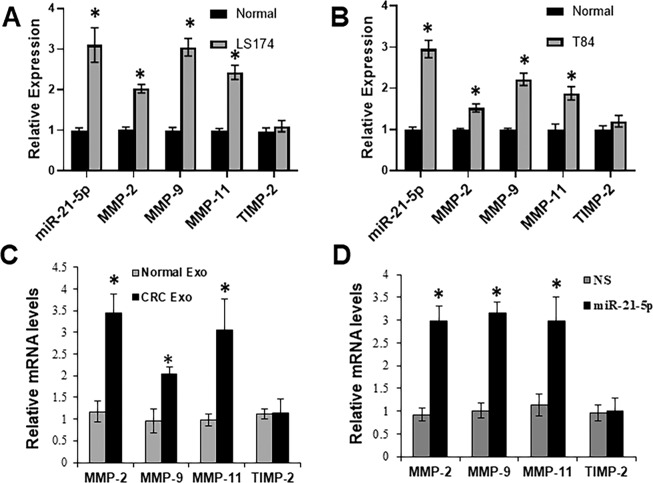
Gene expression of miR-21-5p and matrix metalloproteinase family in Colon cancer cells. The expression levels of endogenous miR-21–5p or MMPs in normal human colon epithelial cell line CRL1831 as compared to (**A**) CRC cell line LS174 or (**B**) CRC cell line T84, as assayed by RT-qPCR (n = 3 individual experiments). (**C**) Cell culture containing 10% FBS of LS174 was treated with equal amounts of exosomes (Exo) purified from serum-free culture media of CRL1831 or LS174 for 24 hr. Then total RNAs were extracted for assessing gene expression levels as indicated (n = 3 individual experiments). (**D**) Cell culture containing 10% FBS of LS174 with was treated with equal amounts (at final concentration of 50 nM) of miR-21–5p mimics or non-specific (NS) control for 24 hr. Then total RNAs were extracted for assessing gene expression levels as indicated (n = 3 individual experiments). *p < 0.01.

As recently shown^14^, miR-21 overexpression caused changes in the expression of ECM mediators in both fibroid and myometrial cells. Therefore, we next determined levels of endogenous miR-21 and ECM mediators in colon cancer cell line LS174 by RT-qPCR, and found a positive correlation between miR-21 and ECM mediators MMP-2, MMP-9, and MMP-11, but not between miR-21 and ECM inhibitor TIMP-2 (Fig. 2A). We further verified our results in another colon cancer cell line, T84, and observed similar trend (Fig. 2B). Again, all MMPs correlated with increase in miR-21 but there was no correlation between miR-21 and TIMP-2.

Nest, we exposed LS174 colon cancer cells with cancer cells or normal cells-derived exosomes and measured mRNA level changes of ECM mediators by RT-PCR. As shown in Fig. 2C, treatment with cancer cells-derived exosomes increased expression of these ECM mediators but not TIMP-2. We also checked with induced expression of miR-21 can have similar effects. To check this, we transfected pre-miR-21 in colon cancer LS174 cells and found a similar increase in MMP-2, MMP-9 and MMP-11 (Fig. 2D), as was seen when cells were exposed to cancer cell-derived exosomes. Collectively, these results suggest that exosomes cause pathological changes in colon cancer cells, at least in-part, by stimulating activity of ECM mediators. Further, miR-21 has similar affects.

### Cancer cells-derived exosomes promote cell proliferation and invasion

**Figure 3:**
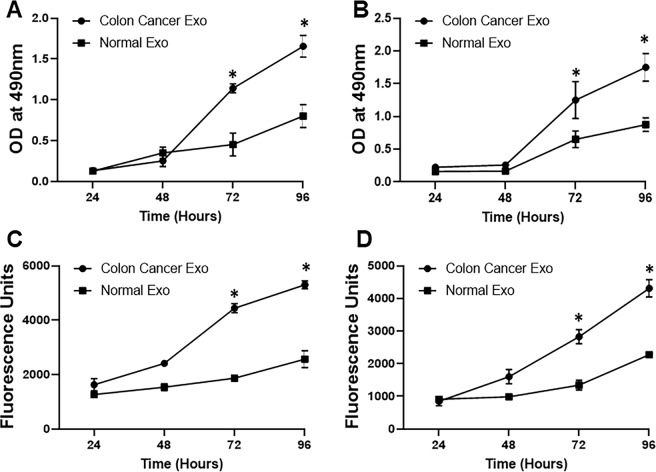
Exosomes from colon cancer cells promote proliferation and invasion. Total exosomes (Exo) were purified from serum-free culture media of CRL1831 (Normal Exo) or colon cancer cells LS174 (Colon Cancer Exo), and then incubated with colon cancer cell lines HT29 (**A**) or T84 (**B**) for the time periods as indicated. The proliferation of colon carcinoma was analyzed by MTT proliferation (n = 3 individual experiments). Invasion of HT29 (**C**) or T84 (**D**) cells was also tested after the addition of exosomes for the time periods as indicated. Cell invasion assay was performed as described in the Methods (n = 4 individual experiments). *p < 0.01.

To investigate the effects of cancer cells-derived exosomes on cell proliferation, human colon cancer cell lines, HT29 and T84, were exposed to exosomes isolated from serum culture media of colon cancer cell line LS174 or the control ‘normal’ CRL1831 cells. When we performed MTT proliferation assay, we found that colon cancer cells (LS174)-derived exosomes promoted cell proliferation (p < 0.01) of both HT29 and T84 adenocarcinoma cell lines (Fig. 3A-B). The proliferation was significantly higher at 3 days (72 hours) and 4 days (96 hours) time points. We also checked for the effects of exosomes on the invasion of cells. Similar to the effects on proliferation, the cancer cells-derived exosomes had a significant (p < 0.01) stimulatory effect on the invasion of both colon cancer cell lines, HT29 and T84, as suggested by the number of cells that invaded through the BME and were detected through fluorescence (Fig. 3C-D). Thus, these results establish that colon cancer cells-derived exosomes induce proliferation as well as invasion, which can have profound effect on cancer metastasis.

### miR-21 promotes CRC cell proliferation

**Figure 4:**
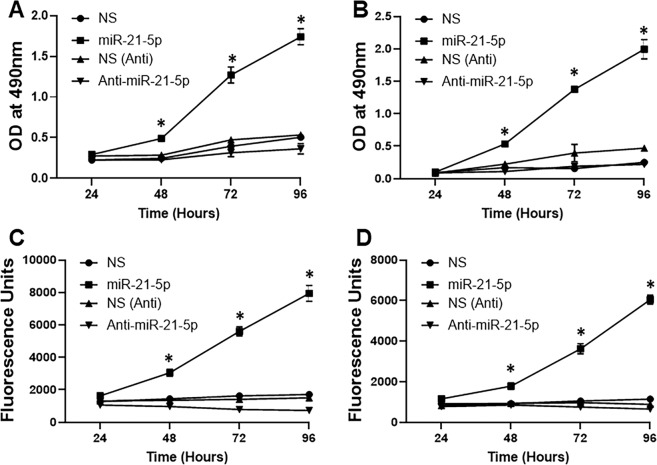
miR-21–5p promotes proliferation and invasion. Colon cancer cell lines HT29 or T84 were transfected with miR-21-5p mimics, anti-miR-21–5p (Anti-miR-21-5p) and their respective control (non-specific, NS or anti-NS) for the time periods as indicated. Proliferation of HT29 (**A**) or T84 (**B**) was analyzed by MTT proliferation assay while invasion of HT29 (**C**) or T84 (**D**) was analyzed by invasion asay (n = 3 individual experiments). *p < 0.01.

To investigate the effect of miR-21 on colon cancer cell growth, HT29 and T84 cells were transiently transfected with miR-21 mimics or miR-21 anatagomirs (Anti-miR-21) and their respective non-specific controls. MTT proliferation assay was performed and the results show that miR-21 mimics promoted (p < 0.01), but miR-21 anatagomirs inhibited cancer cell growth in both colonic adenocarcinoma cell lines, compared to their respective controls (Fig. 4A,B). Similar results were obtained when we assayed for invasion potential. miR-21 mimics significantly (p < 001) promoted cell invasion whereas invasion was reduced by miR-21 antagomirs (Fig. 4C,D). Collectively, these results suggested that miR-21 acts as an oncogenic miRNA to promote cancer cell proliferation as well as invasion of colon cancer cells.

### Exosomes suppress miR-21-target genes

**Figure 5:**
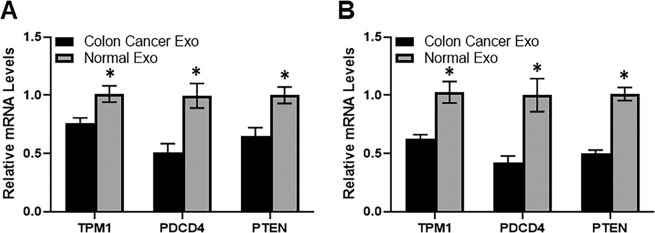
Exosomes from colon cancer cells affect expression of miR-21 target genes. Total exosomes (Exo) were purified from serum-free culture media of CRL1831 (Normal Exo) or colon cancer cells LS174 (Colon Cancer Exo), and then incubated with colon cancer cell lines HT29 (**A**) or T84 (**B**) for 24 hours. Then total RNAs were extracted for assessing gene expression levels of miR-21-target genes, TPM1, PDCD4 and PTEN (n = 3 individual experiments). *p < 0.01.

Since, exosomes affected colon cancer cells similar to miR-21 transfections, we next tested whether exosome effects were because of miR-21 in the cargo. To check this, we looked for the levels of few well-known targets genes of miR-21 after exposing HT29 and T84 colon cancer cells to exosomes derived from LS174 cells, compared to exosomes derived from normal colon epithelial cells. The shortlisted genes, tropomyosin 1 (TPM1), programmed cell death protein 4 (PDCD4) and phosphatase and tensin homolog (PTEN) are reported targets of miR-21^25–28^. We observed that exosomes from cancer cells resulted in significantly (p < 0.01) decreased expression of all of three miR-21 target genes in both of the colon cancer cell lines, HT29 and T84 (Fig. 5). Based on this observation, we conclude that exosomes from colon cancer cells carry miR-21 and, therefore, the expression levels of miR-21 target genes are lower in cells that are treated with these exosomes.

### Silencing of miR-21 target gene mimics miR-21 transfection

**Figure 6:**
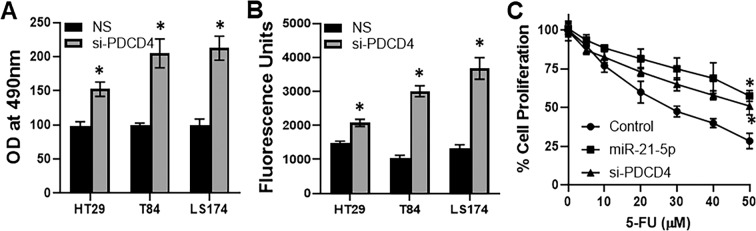
Silencing of miR-21 target gene affects proliferation, invasion and drug sensitivity of colon cancer cells. Colon cancer cells HT29, T84 and LS174 were treated with non-specific control (NS) or si-RNA to silence PDCD4. (**A**) The proliferation of colon carcinoma was analyzed by MTT proliferation assay while (**B**) invasion of cells was performed by invasion assay (n = 3 individual experiments). (**C**) LS174 cells were transfected with miR-21-5p mimics or si-RNA against PDCD4 before treatment with increasing doses of 5-FU for 72 hours and then evaluation of cell proliferation by MTT proliferation assay (n = 3 individual experiments). *p < 0.01.

We observed that exosomes-treatment down-regulated miR-21 target genes and the most significantly down-regulated gene target of miR-21 was PDCD4. Therefore, we next checked if silencing of PDCD4 could mimick miR-21 effects. We first silenced PDCD4 in all of the three colon cancer cell lines, HT29, T84 and LS174 and observed the siRNA to be very effective in down-regulating PDCD4 levels (result not shown). When we checked proliferation, silencing of PDCD4 resulted in significantly increased proliferation (Fig. 6A). Silencing of PDCD4 also resulted in significantly increased invasion (Fig. 6B). Further, we treated control, miR-21-5p mimics/si-PDCD4-transfected LS174 cells with increasing doses of common chemotherapeutic drug 5-FU for 3 days (72 hours) and found that miR-21 significantly induced resistance to 5-FU and similarly silencing of PDCD4 also increased resistance to 5-FU. These results suggest that miR-21–5p functions through the targeted silencing of its target, PDCD4.

## Discussion

In recent years, the value of miRNAs as mediators of various steps of oncogenesis has been realized. Here we report for the first time an important role of exosomal miR-21 in colonic tumorigenesis through stimulation of multiple ECM mediators, promoting cancer proliferation. Our findings have expanded the understanding of the possible functional significance of miR-21 in the pathobiology and genesis of CRC.

microRNAs are an abundant class of endogenous small RNA molecules which bind to target mRNAs and destabilize them^29–31^. They can act as oncogenic or tumor suppressor. It is now well-regarded that miRNAs play an important role in onset and/or progression of many different human cancers, such as, breast, prostate, lung, pancreatic, glioma, meningioma, CRC etc. Oncogenic miRNAs are over-expressed in cancer tissues, compared to normal tissues, where they inhibit apoptosis and promote proliferation, metastasis and resistance to therapy. Tumor suppressor miRNAs, on the other hand, are silenced or under-expressed in cancer tissues, where they function in exact contrast to oncogenic miRNAs i.e. they inhibit proliferation, metastasis and resistance to therapy while supporting induction of apoptosis. An example of tumor suppressor miRNAs is the let-7 family or the miR-200 family^32^. Based on these reported roles of miRNAs, it can be expected that they will one day be used for diagnosis or therapy of human cancers.

There is evidence in support of an epigenetic regulation of miR-21 that distinguishes miR-21 levels in cancer vs. normal tissues^11^. Methylation leads to suppression of expression and, therefore, it makes sense that there exists an inverse relationship observed between expression of miR-21 and its methylation. Because *de novo* DNA methyltransferase DNMT3B is often silenced in CRC cell lines and primary CRC tumors, as a consequence of aberrant DNA hypermethylation of its distal promoter^33^, we postulated that increased expression of miR-21 in CRC may be a consequence of DNMT3B activity, and DNMT3B is likely responsible for hypermethylation of miR-21, which usually occurs in healthy colon tissues.

miRNAs negatively regulate their target genes. Accordingly, we found that miR-21 increased expression of multiple ECM mediators in CRC. Obviously, miR-21 up-regulates ECM mediators most likely in an indirect manner. We also report that several target genes of miR-21, such as, PDCD4, TPM1 and PTEN are down-regulated by exosomes from colon cancer cells, and, further, silencing of PDCD4 mimics the effects of miR-21 transfections. Furthermore, miR-21 can mediate resistance to therapy such as 5-FU through down-regulation of PDCD4. To sum up, here we report that exosomes of CRC have profound effect on proliferation, invasion and even drug resistance. We further show that exosomal miR-21 promotes CRC cell growth through stimulation of activity of ECM mediators. Based on these findings, it will sense to speculate that targeting of exosome secretion can be an effective anti-cancer strategy. Future research should be focused on means to accomplish this for the benefit of CRC patients who have exhausted other modes of treatment.

## Data Availability

All data is presented within the article.
